# Turning over New Leaves

**Published:** 1889-03-09

**Authors:** 


### Turning Over New Leaves.

(Books for Review should be sent to The Editor. The Lodge, Porchester Square, W.)

### A Life Register.*

"Know thyself," said the Delphic sage. Preachers in all ages have been telling us how imperfectly we have obeyed this dictum on the moral side; but it has been left to our generation to come to the conclusion that it is well to know oneself physically too. This little book renders both easy and inviting such a record of ourselves as would not only be interesting to the subjects of it, but would be of considerable value to our doctors. A physician has always to spend a good deal of time in finding out the antecedents and life-history of new cases, and very often the record obtained by questioning the patient or his friends is neither accurate nor satisfactory. While illness was looked upon as a spontaneous development, a pathological Topsy, the doctor's work was only to treat symptoms as they arose; but those days are gone. Now he inquires for constitutional predispositions, and sees in diseases that arise without apparent causes the *sequelæ* of forgotten illnesses. Therefore, a patient who can present a clear and compact history of his physiological career facilitates the doctor's task and helps his own recovery. For such a history this register is admirably adapted. In the front there are blank pages left for photographs of parents. Then come pages to be filled with the health record of each succeeding year, with specified questions to be answered by parents during the youth of the subject of the register, showing the details of growth and development. The register goes on to the seventy-second year, and the concluding pages are occupied with sound and simple hygienic instructions. Both as a matter of curiosity and forethought, this little book is better worth having and filling than most diaries.

### Some Social Types.*

This book consists of a series of clever sketches of personages we all come across more or less in daily life, not caricatures, yet sharply touched with satiric humour. The tennis-playing amazon, the flirting grass widow, the lugubrious lady novelist, the man-of-the-world who has not yet completed his eighteenth year, the shady financier, and the ritualistic curate—these—the other types in the book we have met in the life, though, perhaps, with their characteristics less marked than in Mr. Parkes's illustrations. We should like to suggest, however, that Sir Digsby Bunkum, M.D., K.C.S.I., etc., author of the "Marvellous Manifestations of a Microcosm," is gloomier than any doctor we ever met; and that the height of Mrs. Agompiler's forehead is something of a physiological miracle. But altogether this is a good and amusing shillingsworth.

* Life Register. London: West, Newman, and Co., 54, Hatton Garden. Price 1s. 6d.

* "People We Meet." By Charles F. Rideal. Illustrated by Harry Parkes. London: Field and Tuer, Ye Leadenhalle Presse. Price 1s.

